# The application of high-performance ultrasound probes increases anatomic depiction in obese patients

**DOI:** 10.1038/s41598-023-43509-9

**Published:** 2023-09-28

**Authors:** Sascha Heinitz, Jürgen Müller, Klaus-Vitold Jenderka, Haiko Schlögl, Michael Stumvoll, Matthias Blüher, Valentin Blank, Thomas Karlas

**Affiliations:** 1 Clinic for Endocrinology, Nephrology, and Rheumatology, Leipzig University Medical Center, Liebigstrasse 20, 04103 Leipzig, Germany; 2 Helmholtz Institute for Metabolic, Obesity and Vascular Research, Philipp-Rosenthal-Strasse 27, 04103 Leipzig, Germany; 3 Department of Diagnostic and Interventional Radiology, Leipzig University Medical Center, Liebigstrasse 20, 04103 Leipzig, Germany; 4 Department of Physics, Sensor and Ultrasound Technology, University of Applied Sciences Merseburg, Eberhard-Leibnitz-Strasse 2, 06217 Merseburg, Germany; 5 Department of Medicine II, Division of Gastroenterology, Leipzig University Medical Center, Liebigstrasse 20, 04103 Leipzig, Germany; 6 Division of Interdisciplinary Ultrasound, Department of Internal Medicine I, Halle University Medical Center, 06120 Halle (Saale), Germany

**Keywords:** Gastroenterology, Health care, Medical research

## Abstract

This study evaluated the impact of obesity on abdominal ultrasound diagnostics and assessed effect of high-performance ultrasound probes increased imaging quality. Lean and obese subjects (n = 40; 58% female) were categorized according to body mass index (BMI, 21 to 48 kg/m^2^). A highly standardized ultrasound examination of the abdomen was performed by trained examiners using three different probes in randomized order (standard probe versus two high-performance probes). Quality of B-mode and duplex ultrasound were assessed using a custom scoring approach for depiction of liver and kidney anatomy and vascularization. Across probes, imaging quality of hepatic and kidney anatomy was inversely related with BMI (*P *< 0.03, r < − 0.35). Age, sex, and BMI explained 51% of the variance within the ultrasound quality score, with β = − 0.35, *P *< 0.0001 for BMI. Compared to the standard probe, high-performance probes allowed for a better depiction of kidney and liver anatomy in subjects above BMI 35 kg/m^2^ (n = 20, all *P *< 0.05), resulting in a less pronounced deterioration of imaging quality with increased BMI (all *P *< 0.05). In conclusion the study shows that obesity impairs ultrasound imaging quality of abdominal anatomy. The application of high-performance probes can increase anatomic depiction in obese patients.

Registration number of the German Registry of Clinical Studies: DRKS00023498.

## Introduction

Ultrasound examination of the abdomen has a central role as a diagnostic tool in patient care and is the recommended first-line approach for many medical conditions^1,2^. Compared to other imaging techniques, i.e. computed tomography (CT) and magnetic resonance imaging (MRI), ultrasound is readily available and, therefore, predestined in point-of-care diagnostics^1^. Moreover, ultrasound avoids unnecessary radiation exposure and can be applied in almost every individual.

Obesity and related disorders, especially hepatic steatosis, represent commonly accepted limitations to ultrasound imaging quality, and, thus, impair its diagnostic value^3–6^. Trained operators may manage these issues by adjustment of ultrasound frequency, gain, and focus settings^7^, but patients with severe obesity remain a challenge even for modern ultrasound devices with advanced post-processing algorithms designed to improve imaging quality.

Novel ultrasound probe technologies aim at higher penetration depths and improved imaging quality in persons with higher-grade obesity^6,8^. The benefit of such technologies, however, has not yet been sufficiently evaluated and appropriate probes are only available in few centers. In addition, there are limited reports on how medical care of patients at risk for severe hepatic steatosis due to obesity or other diseases (e.g. type 2 diabetes) are affected by insufficient ultrasound visualization^9^. Nevertheless, current national and international guidelines for fatty liver disease and diabetes recommend abdominal ultrasound not only for the diagnosis of hepatic steatosis, but also as a regular screening tool for complications such as hepatocellular carcinoma^10–12^, albeit a high sensitivity is of utmost importance in such scenarios. Independent reports indicate that one out of five ultrasound examinations in patients with cirrhosis, and particularly with obesity, are inadequate for exclusion of hepatocellular carcinoma^13^.

Recently, the question of imaging performance relative to ultrasound penetration depth was addressed in an experimental approach: Using a phantom, multiple transducers were investigated regarding resolution at different depths^14^. In this setting, matrix probes performed best. Data on the impact of high-performance probes on imaging quality in patients, however, is scarce. A recent pilot study revealed differences between curved array transducers, but did not analyze the impact of body size on imaging quality^15^.

The present study aimed to assess the impact of obesity and hepatic steatosis on the imaging quality and clinical utility of abdominal ultrasound. The study aim was investigated using a head-to-head comparison of a standard abdominal probe versus two high-performance probes with different signaling technology in subjects with normal body weight versus overweight and obesity.

## Patients and methods

In a standardized monocentric, randomized approach, the current prospective study investigated whether obesity relates to impaired ultrasound assessment of abdominal anatomy in subjects of different body mass index (BMI, range 21–47.7 kg/m^2^) and whether this is affected by probe performance. In brief, study participants were randomized to a predefined sequence of ultrasound examinations of the liver and right kidney using three different probes (standard versus high-performance probes). Imaging quality was assessed using a highly-standardized custom ultrasound score (see below).

To address differences in imaging quality using the standard versus high-performance probes in a patient-independent approach, targets within liver phantoms were investigated in the present study, as well.

The study was approved by the Ethics Committee of the Medical Faculty of the University of Leipzig (485/20-ek) and registered at the German Registry of Clinical Studies (DRKS, register number DRKS00023498). The study was performed in accordance with the guidelines for good clinical practice (E6/R1) and the ethical guidelines of the Helsinki Declaration. All participants provided written informed consent.

### Ultrasound probes

All examinations were performed using two high-performance ultrasound devices (Acuson Sequoia, Siemens Healthineers, Erlangen, Germany) and Canon Aplio i800 (Canon Medical Systems, Ota, Japan). Three convex-shaped probes, a conventional standard probe (SP, 5C1 Acuson Sequoia, Siemens Healthineers) and two high-performance probes (HPP) 1 (Deep abdominal transducer, DAX, Acuson Sequoia, Siemens Healthineers) and 2 (Ultra-Wideband Matrix Convex i8CX1, Aplio i800, Canon) were included in the current study to test for differences in ultrasound imaging quality. The applied probes use different technical improvements to enable a higher penetration depth in obese patients. HPP1 will add crystal elements depending on the depth of the area of interest and HPP2 uses a single crystal low-noise transducer array technology in combination with a matrix array transducer element design to achieve high signal-to-noise ratio especially in the far field. For a detailed description of used probes, please refer to Supplementary Table 1 and Supplementary Fig. 1.

### Liver phantoms

To address the issue of differences in imaging quality using the standard versus high-performance probes in a patient-independent setting, the visualization of targets within commercially available ultrasound phantoms were investigated in a non-clinical approach.

In a first step, to evaluate (a) the capability of discrimination of focal lesions of defined sizes and depths, and (b) spatial resolution and measurement precision, SP vs. HPP1 and HPP2 were used in two multi-purpose ultrasound phantoms with similar acoustic characteristics compared to human tissues. A detailed description of phantoms used can be found in the Supplement.

### Ultrasound examination and ultrasound quality scores

To address the issue of quality assessment of ultrasound examination of the abdomen in a clinical approach, specifically of the liver and right kidney, a custom ultrasound score was created that aimed to assess overall quality of investigation of liver and kidney (i.e. total ultrasound score) adding to 33 points for complete imagining of addressed items during examination. The total ultrasound score consisted of two sub-scores for liver anatomy (i.e. liver ultrasound score, maximum value 25 points) and anatomy of the right kidney (i.e. kidney ultrasound score, maximum value 6 points). Visualization of hepatic and kidney vascular anatomy entered a separate score, i.e. vascular ultrasound score (maximum value 17 points). A detailed description of investigated structures and how ultrasound scores were calculated can be found in the Supplement (Supplementary Table 2).

### Study participants

Prior to study enrolment, written informed consent was obtained from all subjects. Study exclusion criteria were pregnancy/lactation, surgery leading to altered hepatic and/or kidney anatomy (e.g. partial liver or kidney resection), invasive laparotomy, ascites, or relevant meteorism. Subjects were assigned to study groups defined by BMI:

Group I, normal weight = BMI 18.5–24.9 kg/m^2^.

Group II, overweight and adiposity stage I = BMI 25–34.9 kg/m^2^.

Group III, adiposity stage II = BMI 35–39.9 kg/m^2^.

Group IV, adiposity stage III = BMI >40 kg/m^2^.

Waist-to-hip ratio (WHR) was measured manually and diabetes status assessed via self-report. Then, in randomized order and using three different transducers (see Ultrasound probes), a highly standardized ultrasound examination of the liver and the right kidney was performed by trained examiners.

### Elastography, liver stiffness measurements, and controlled attenuation parameter

Transient elastography allowed for assessment of liver stiffness (in kPA). Elastography measurements were performed immediately before or after the ultrasound study examinations following general recommendations for liver stiffness measurements^16^. As elastography reference, liver stiffness (in kPa) and controlled attenuation parameter (CAP, in dB/m) were measured using a Fibroscan Compact 530 device. For details, please refer to the Supplement.

### Statistical analysis

Data were analyzed using GraphPad Prism (Version 9.0; San Diego, CA, USA) and SAS Enterprise Guide (Version 7.13; Cary, NC, USA).

Subject characteristics were compared using the Mann Whitney U test. The Friedman test compared differences between ultrasound assessment using SP, HPP1, and HPP2 in the current paired design with post test results allowing for interpretation of directionality. Within-probe performance was tested via the Kruskal-Wallis test with post testing to interpret effect sizes. Post tests were adjusted for multiple comparisons. Linear regression tested dependency of measures of body size relative to ultrasound scoring. General alpha-level was set at 0.05.

One-way-ANOVA was used to test for differences in CAP between BMI groups. The cutoff for optimal visualization of the liver and right kidney as performed using the total ultrasound score was set at 32 points via calculating the mean score achieved within BMI group I, in a setting of poorer imaging quality for BMI groups II through IV across probes (unpaired *t* test: *P *< 0.0001; − 7.82 points, 95% confidence interval − 7.82±1.40 points).

## Results

### Liver phantoms

**Figure 1:**
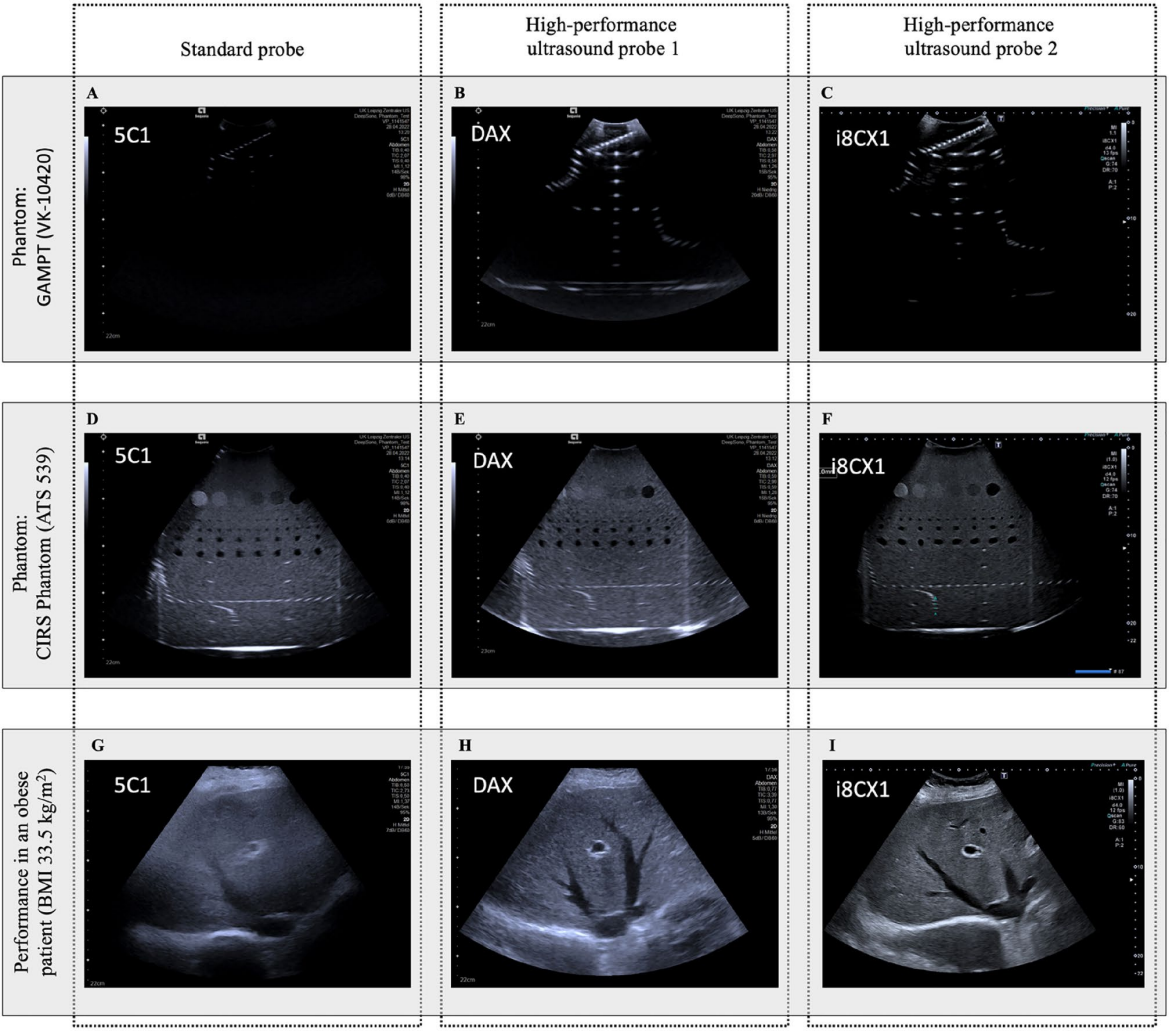
Comparison of two phantoms and application in an obese patient using the standard probe versus two high-performance probes. (**A**–**C**) Comparison of the performance of the three applied ultrasound probes in a standardized ultrasound phantom with high attenuation of the ultrasound signals (GAMPT VK-10420). (**D**–**F**) Comparison of the three ultrasound probes using another ultrasound phantom. (**G**–**I**) Illustration the application of standard probe and the two high-performance ultrasound probes in an obese patient (BMI 33.5 kg/m^2^).

**Figure 2:**
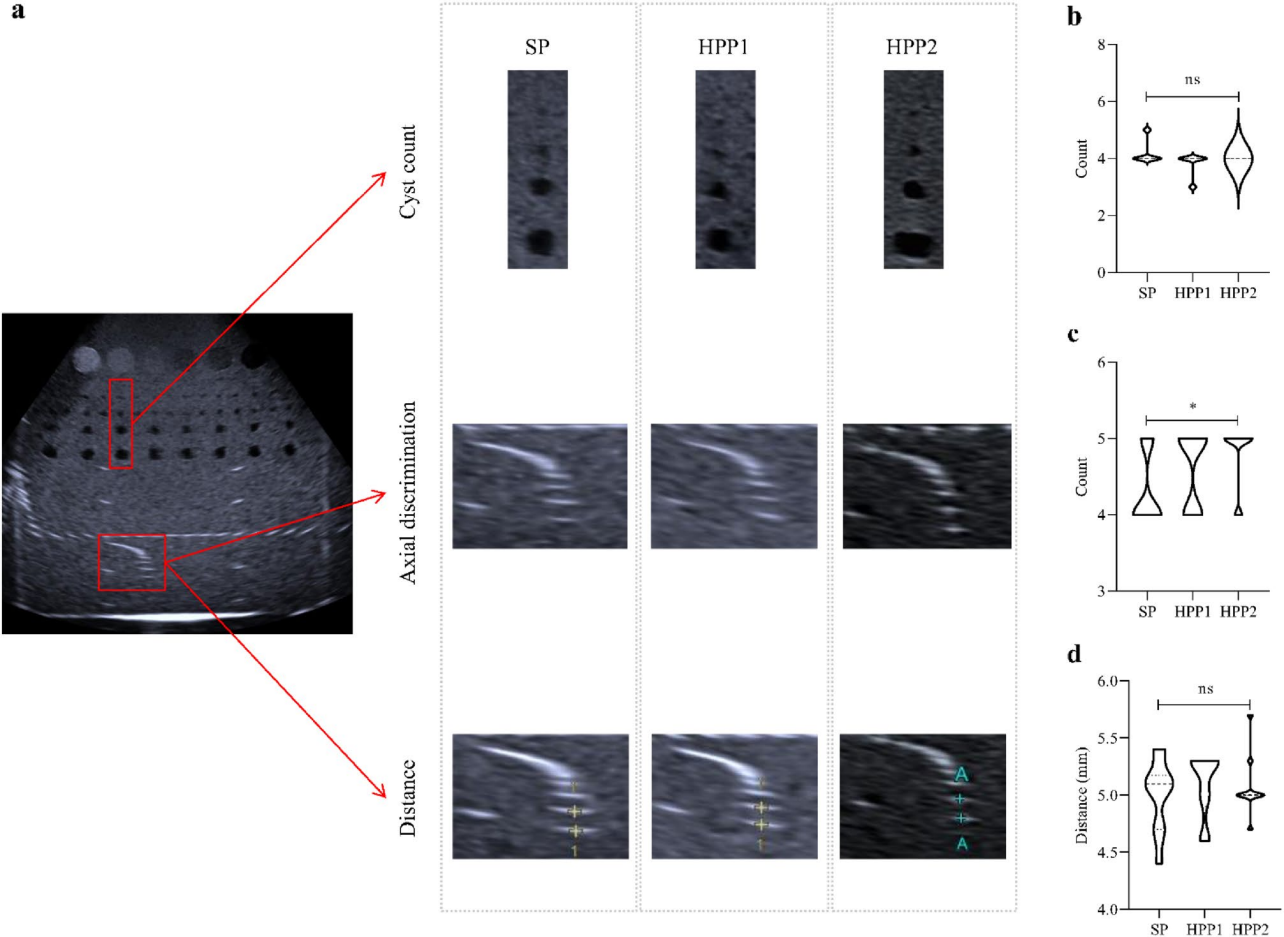
Comparison of target detection using the phantom 2. Targets consisted of up to 5 cysts within 65 to 108 mm depth, a group of up to 11 dots within 153 to 172 mm depth for axial discrimination (**a**). Cyst count (**b**), as well as distance discrimination (**d**) did not differ comparing SP to high-performance probes. SP had a lower axial discrimination compared to the latter (**c**). Median indicated by a dashed line, quartiles indicated by a dotted line. *P < 0.05. *HPP* high-performance probe, *ns* not significant, *SP* standard probe.

Liver phantoms as investigated using SP, HPP1, and HPP2 are shown in Fig. 1. Targets within phantom 1 were poorly visualized by SP with only moderate differences comparing HPP1 to HPP2. Within phantom 2 (Fig. 2a), cyst count (Fig. 2b) and distance (Fig. 2d) did not differ comparing SP to HPP1 and HPP2. Axial discrimination was lower in SP versus HPP1 and HPP2 (*P*<0.05, rank sum difference − 4.5 and − 7.5, respectively, Fig. 2c).

### Patients’ characteristics and ultrasound scoring system

Subject characteristics are shown in Table 1. Comparing group I to groups II through IV, skin-to-liver distance and CAP were different (all *P* < 0.01), whereas WHR and liver stiffness did not differ (all *P* > 0.05). No subject within group I was diabetic. Assessment of imaging quality of liver and right kidney anatomy as calculated using the custom scoring system and relative to BMI groups is shown in Table 2.

**Table 1:** Subject characteristics.

Subject characteristics	BMI (kg/m^2^) group I–IV			
	(I) 18.5–24.9	(II) ≥25–34.9	(III) ≥35–39.9	(IV) ≥40
N	10	10	10	10
Female (%)	60	40	60	70
Age (years)^†^	28 [25–31]	36 [30–51]	44 [35–62]	46 [34–56]
Type 2 diabetes (%)	0	10	50	30
BMI (kg/m^2^)^†^	22.6 [21–23.9]	30 [27–31.9]	37.8 [36.5–38.8]	44.5 [42.6–47.7]
Waist-to-hip ratio^†^	0.79 [0.75–0.87]	0.98 [0.8–1.05]	0.91 [0.85–0.97]	0.91 [0.82–0.95]
Skin-to-liver distance (mm)^†,#^	14 [12–15]	21 [20–23]	28 [23–33]	36 [33–44]
Liver stiffness (kPa)^†,§^	4.9 [4.2–5]	4.5 [3.7–6]	4.9 [3.8–6.8]	6.3 [4.2–9.6]
Controlled attenuation parameter (dB/m)^†,§^	198 [157–217]	263 [220–310]	311 [286–327]	349 [348–362]

*BMI* body mass index, *LSM* liver stiffness measurement.

^†^Median, inter-quartile range, ^#^At LSM measuring site, ^§^n = 33 valid measurements (n = 3 within group III, n = 4 within group IV).

**Table 2:** Depiction of liver of kidney anatomy.

	BMI (kg/m^2^) group I–IV			
	(I) 18.5–24.9	(II) ≥25–34.9	(III) ≥35–39.9	(IV) ≥40
Total ultrasound score				
SP	32.5 [31.25–33]	27 [16.5–31.75]	24 [17–30.75]	23.5 [14.5–24.75]
HPP1	32 [31–32]	28.5 [25.25–32]	26 [21.75–31.5]	25 [21–29.75]
HPP2	33 [32–33]	29 [26–31.75]	27.5 [22.5–31.75]	25.5 [16.25–28.75]
Liver ultrasound score				
SP	24 [23–24]	19 [10.75–23.75]	16.5 [11.25–23]	17 [10.25–18]
HPP1	23.5 [23–24]	21 [17.5–23.75]	20 [16.25–22.75]	18.5 [14.75–22.75]
HPP2	25 [24.25–25]	22 [19–24.75]	21 [16.75–23.75]	21 [13.5–23]
Kidney ultrasound score				
SP	6 [5.25–6]	5 [4–6]	4 [3.25–5.75]	3 [2.25–3.75]
HPP1	6 [5–6]	5 [4.25–5.75]	5 [4–6]	4 [4–4]
HPP2	6 [6–6]	5 [4–6]	5 [3.25–6]	3 [2–3.75]
Vascular ultrasound score				
SP	16 [15.25–17]	15 [8.5–15.75]	11.5 [6.25–15]	10 [5–11]
HPP1	16 [15.25–16]	15 [12.25–16]	13 [10.5–15.75]	11.5 [8.5–13.75]
HPP2	17 [16.25–17]	15.5 [13–16]	14 [8.75–15.75]	11 [7–12.75]

The total ultrasound score consisted of liver, kidney, and vascular (hepatic and kidney) scoring. Median and inter-quartile ranges shown. Applying a CAP-cutoff for steatosis grade 1 = 302 dB/m, grade 2 = 331 dB/m, and grade 3 = 337 dB/m^17^, n = 27 subjects had no sign of steatosis, whereas n = 5 subjects had grade 1 steatosis, n = 2 subjects had grade 2 steatosis, and n = 6 had grade 3 steatosis. CAP (mean 261.9 dB/m, SD 74.4 dB/m) increased with greater BMI (*P* < 0.0001, r = 0.62) and correlated with BMI (*P* < 0.0001, r = 0.80). Four subjects (BMI ≥ 36.3 kg/m^2^) displayed signs of fibrosis, according to Fibroscan (cutoff 8 kPa)^11^.

*HPP* high-performance probe, *SP* standard probe.

### Ultrasound examination is affected by probe performance

**Figure 3:**
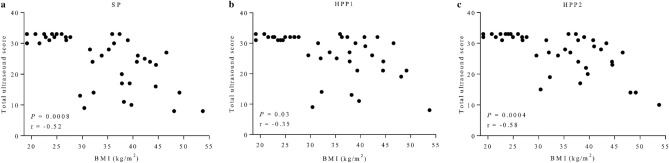
Correlation of body mass index with total ultrasound score. Depiction of liver and kidney anatomy, as assessed using a custom ultrasound score, deteriorates with greater BMI. Pearson correlation reported. *BMI* body mass index, *HPP* high-performance probe, *SP* standard probe.

**Figure 4:**
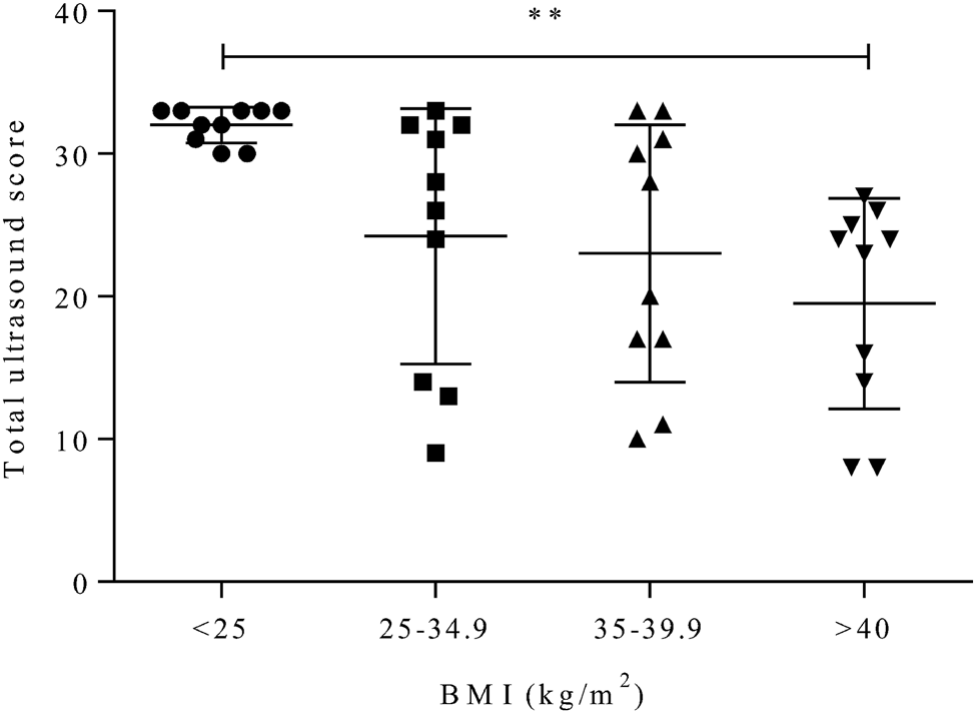
Ultrasound scoring for the standard probe across BMI groups. Visualization of liver and kidney anatomy decreased across BMI groups and with higher BMI (all P < 0.01). *BMI* body mass index, *SP* standard probe.

**Figure 5:**
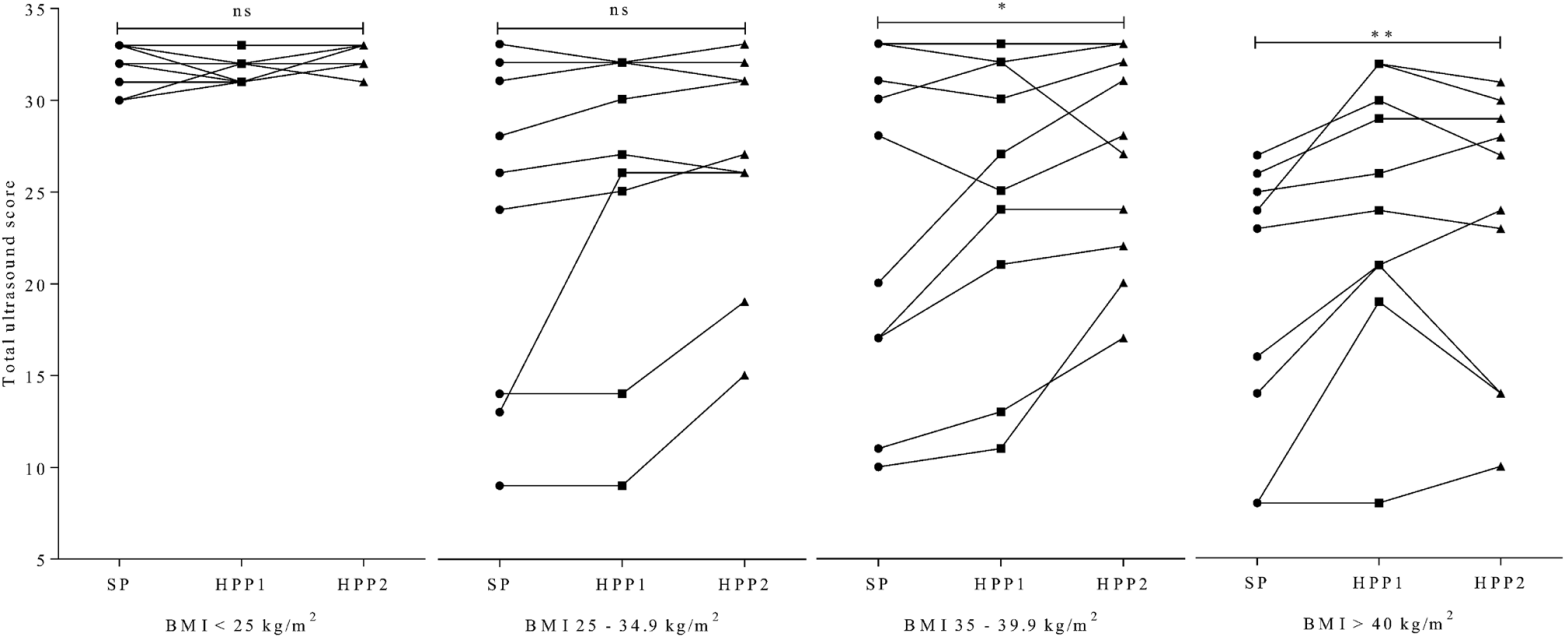
Improved assessment of liver and kidney anatomy in subjects with greater adiposity. Relative to SP, no difference in ultrasound imaging was detected for subjects with BMI < 35 kg/m^2^. In subjects with BMI ≥ 35 kg/m^2^ total ultrasound imaging scored higher when using the high-performance probes and relative to SP (all P < 0.05). *BMI* body mass index, *HPP* high-performance probe, *ns* not significant, *SP* standard probe.

As measured using the total ultrasound score, assessment of liver and kidney anatomy deteriorated with increases in BMI, irrespective of ultrasound probe (all *P* < 0.03, r < − 0.35; Fig. 3a–c). As indicated by the inverse correlation as shown in Fig. 3a–c, across probes, subjects with BMI < 25 kg/m^2^ scored higher compared to other BMI groups. However, for certain subjects with BMI ≥ 25 kg/m^2^ total ultrasound score was comparable to normal-weighed individuals. Notably, for HPP1 and HPP2, but not SP, this was true for subjects with BMI > 40 kg/m^2^. Total ultrasound score, as assessed using SP, decreased across BMI groups (*P* = 0.002, mean rank difference > + 13.1; Fig. 4). Relative to SP and within BMI groups, high-performance probes reduced deterioration of imaging quality as they allowed for a more complete depiction of kidney and liver anatomy in subjects above BMI 35 kg/m^2^: In subjects with BMI 35–39.9 kg/m^2^, scoring differed across probes (*P* < 0.05, Fig. 5) with differences driven by a tendency for a more complete depiction using HPP2 (*P* = 0.06). In subjects with BMI ≥ 40 kg/m^2^ and relative to SP, total ultrasound scores were different across probes (*P* *=* 0.001), with differences for both high-performance probes in post-hoc analyses (for HPP1 *P* = 0.004, rank sum difference = – 14; for HPP2 *P* = 0.05, rank sum difference = – 10).

Across overweight and obese subjects and relative to SP, imaging quality of liver improved with implementation of high-performance probes (*P* < 0.007, rank sum difference < – 4), thus indicating that imaging quality of liver did not depend on the degree of obesity but was entirely dependent on probe performance. Notably, except for BMI >40 kg/m^2^, where use of HPP1 and HPP2 both lead to better depiction of liver anatomy (Dunn’s test: all *P* < 0.05), improved performance in subjects with BMI 25–39.9 kg/m^2^ was driven by HPP2 (all *P* < 0.05). Fig. 1 provides an example for the visual difference in ultrasound imaging quality of the liver in an obese subject.

For kidney ultrasound score, imaging quality did not differ comparing SP with HPP1 and HPP2, irrespective of BMI group (all *P* > 0.05). However, relative to SP, subjects with BMI ≥ 40 kg/m^2^ tended to a better imaging quality (*P* = 0.07). Comparable to the total ultrasound score, high-performance probes allowed for greater imaging quality regarding vascularization and relative to SP in subjects with BMI ≥40 kg/ m^2^ (*P* < 0.0001, rank sum difference < – 13), but not for subjects within BMI groups I through III (all *P* *>* 0.05).

### Analysis of factors associated with ultrasound quality

In multivariate analyses and across probes, up to 51% of the variability within the total ultrasound score was accounted for by age, sex, and BMI, with β < − 0.47 score ranking for BMI (*P* < 0.03). For HPP1 and HPP2, but not for SP, WHR was a determinant for total scoring in a model including age, sex, as well, explaining up to 52% of the variance within the score, with β < − 15 score ranking for WHR alone (*P* < 0.03). In these models, WHR and BMI were independent determinants (all *P* < 0.05). Irrespective of BMI and WHR, CAP was a determinant of imaging quality for the total ultrasound score (*P* < 0.05, β < − 0.06 score ranking) when introduced into the model with age and sex. Variability explained by CAP, however, was mediated by BMI (all *P* > 0.05 for CAP upon inclusion of BMI into multivariate analyses).

### Agreement of metric measurements

To study the metric agreement within the three different probes, we evaluated the size of the right kidney (longest diameter).

**Figure 6:**
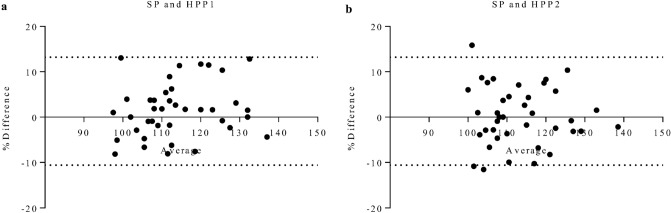
Bland-Altman comparisons of standard probe versus high-performance ultrasound probes. Differences in organ size assessment do not tend to differ with changes in average organ size comparing SP versus HPP1 (**a**) or HPP2 (**b**). There are no changes in variability between the two probes, displaying visually equal average estimations across measured organ size. *HPP* high-performance probe, *SP* standard probe.

In a Bland-Altman comparison, average bias of SP versus HPP1 was 1.3 mm (standard deviation, SD, ± 6 mm), with 95% of the differences between these probes ranging from − 11 to 13 mm. Thus, clinically, discrepancy in kidney size estimation was low with narrow limits of agreement regarding total organ size. Differences in organ size estimation between the two probes visually do not differ relative to average kidney size, displaying consistent variability (Fig. 6a). Comparing SP versus HPP2, the same was true: Average bias was 0.12 mm (SD, ± 6.4 mm), displaying even lower bias regarding organ size estimation. Ninety-five percent limits of agreement with respect to kidney size were − 12 mm and 13 mm (Fig. 6b): Comparing SP and HPP2, estimation of kidney size did not differ, as variability across average kidney size was visually the same in here performed Bland-Altman comparison.

### Degree of steatosis and agreement of fibrosis

In addition to B-Mode ultrasound morphology and metric measures, the standardized analysis of tissue characteristics represents an important aspect of liver ultrasound examination. Therefore, we compared results of steatosis and liver stiffness measurements across probes.

Conventional B-Mode-based scoring for the degree of steatosis differed across probes (*P* = 0.03), with a trend to attribute greater fat infiltration to subjects for HPP2 (rank sum difference = + 9) versus comparable scoring using HPP1 (rank sum difference = − 1.5; Supplementary Fig. 2) compared to SP, respectively.

Regarding liver stiffness measurement, shear-wave elastography as performed using HPP1 (*P* = 0.0021, r = 0.27) and HPP2 (*P* = 0.03, r = 0.15) correlated with measurements obtained with SP, respectively (Supplementary Fig. 3a and b). Within a range of 4 to 7 kPa, however, all probes performed poorly regarding their ability to correspond to fibrosis as detected using gold standard Fibroscan assessment (Supplementary Fig. 3c).

### Clinical aspects

At the end of the examination of the liver, the investigators had to state whether it would have been likely to detect a hepatic lesion ≥ 1 cm with the probe used (SP, HPP1, or HPP2). Probes performed differently in their ability to assure detection of such a lesion (*P* = 0.01) across investigated BMI groups, however, with no differences comparing HPP1 and HPP2 to SP in post-hoc analyses, respectively (all *P* < 0.05).

Optimal imaging quality for the total ultrasound score (scoring ≥ 32 points) was achieved by subjects of comparable body size (BMI and WHR) and steatosis (CAP) across probes (Supplementary Table 3). There were no considerable differences in BMI, WHR, and CAP in subjects with non-optimal imaging quality (scoring < 32 points). Taken together, imaging quality deteriorated with the onset of more than moderate overweight and WHR. In line with above mentioned finding that CAP is not a significant determinant of total ultrasound imaging quality, individuals with non-optimal imaging quality had comparable CAP scores below steatosis grade 1^17^.

## Discussion

The present study compared two high-performance ultrasound probes with a standard probe in standardized phantoms and study participants to detect differences in imaging quality relative to anthropometric measures. Imaging quality was defined as (i) in-depth assessment of targets within studied phantoms and (ii) the ability to investigate a multitude of anatomic structures of the liver and right kidney aiming at a complete visualization of these organs. In our non-clinical approach, high-performance ultrasound probes achieved a higher axial resolution in a standardized ultrasound phantom. In the clinical investigation, as expected, assessment of liver and kidney anatomy deteriorated with greater BMI irrespective of probe type and transducer technique. For high-performance ultrasound probes, BMI and WHR were independent determinants of ultrasound quality scoring. Implementation of high-performance ultrasound probes in the depiction of liver and kidney anatomy reduced deterioration of imaging quality relative to the standard probe as it lead to a more complete assessment in subjects with BMI above 35 kg/m^2^ with a pronounced effect in subjects with BMI greater 40 kg/m^2^. However, in the majority of patients with severe obesity, none of the probes achieved a high imaging quality comparable to lean subjects.

It is well accepted that obesity constitutes a challenge for the utilization of ultrasound in medicine^3–5^, however, there is little data on its impact on the abdominal examination^6,8^, with, to our knowledge, no standardized, prospective, and randomized approaches published studying the clinical impact of BMI on imaging quality. Ultrasound examination of subjects with severe obesity is particularly difficult, as adjustments of ultrasound settings may not diminish the impact of body size on imaging quality^7^. Aiming at a greater in-depth visualization, implementation of high-performance ultrasound probes in ultrasound assessment of subjects with (severe) obesity therefore constitutes a promising approach in the attempt to provide high quality diagnostic standards^6,7^. To add to the field of abdominal ultrasound examination and the effect of obesity on imaging quality, we here performed a clinical trial addressing the impact of BMI on the depiction of liver and kidney anatomy using a custom ultrasound quality score.

Our results are in line with common expert opinions and clinical studies investigating non-abdominal ultrasound in subjects with obesity, i.e. that obesity impairs the accuracy of abdominal ultrasound diagnostics^18,19^. In the current trial, this is especially true for subjects with BMI above 35 kg/m^2^. Therefore, examination results from cohorts with severe obesity need to be interpreted carefully and alternative modes of imaging (i.e. computer tomography, magnetic resonance imaging (MRI)) must be considered^19,20^. Nevertheless, patients with severe obesity also suffer from limitations using other imaging techniques (e.g. restrictions of the MRI gantry diameter). Notably, irrespective of the degree of obesity/overweight, in the current trial assessment of liver anatomy was entirely dependent on probe performance, pointing to the value of enhanced in-depth visualization using modern ultrasound techniques.

The here observed stepwise deterioration of imaging quality with higher BMI classes underlines the need for a definition of a diagnostic minimum imaging quality standard, especially for the examination of the liver. Given the obesity pandemic in industrialized countries, this is of utmost importance, as national and international clinical guidelines recommend abdominal ultrasound as the method of choice for screening and observation of patients with fatty liver disease at risk for liver cirrhosis and hepatocellular carcinoma^11,12^. Regarding the assessment of focal lesions, however, although our data point to a difference in the confidence of an investigator to detect a lesion within the liver across probes and BMI groups, our study was likely not powered to address the question, whether a high-performance ultrasound probe drove this effect. Above mentioned overall improved imaging quality within liver related to the use of high-performance ultrasound probes, however, indicates that differences in investigator confidence need to be attributed to the probe used.

Although imaging quality of liver and kidney anatomy deteriorates with greater BMI, the current data show that BMI alone is an insufficient predictor of imaging quality. Therefore, age, sex, anthropometry such as WHR, and risk of high degree steatosis should also be considered before a patient is scheduled for ultrasound diagnostics to allow for optimal interpretation of images obtained during examination – or, depending on the question the investigation aims to answer, whether ultrasound is the right technique to use^21^. Moreover, the observed differences between a standard ultrasound probe and the high-performance probes raise the question if existing technical quality requirements^22^ are sufficiently high for patients with metabolic disease. Potentially, exclusive use of high-performance ultrasound probes can improve the success of screening and observation programs. This should be addressed in further prospective clinical trials.

Possibly due to differences in post-processing of achieved image data using different ultrasound engines, in the present study the two high-performance ultrasound probes differed in regard to estimating the degree of steatosis relative to the standard probe. Liver stiffness measurement was comparable across probes, however, corresponded poorly with Fibroscan. This may be explained with a relatively small amount of subjects with liver stiffness within the range >7 kPa, thus indicative of higher-degree liver fibrosis.

For vascularization, the effect of high-performance probes was comparable to those as observed for liver and kidney anatomy depiction. For the right kidney, however, there was no benefit in addressing kidney anatomy using the standard versus high-performance probes. Previously, kidney depth was found to be a suitable predictor for good contrast-enhanced ultrasound in subjects with obesity^23^. Kidney depth was not investigated in the present study, thus potentially did not differ significantly across overweight/obese subjects. This is supported by our Bland-Altman analyses visually pointing to comparable kidney size estimation across probes.

It needs to be noted, that improvements of spatial resolution in ultrasound imaging have let to better clinical application and patient care before: High-resolution linear probes have added to the field of breast ultrasound allowing for better axial and lateral resolution, and tissue contrast, among others, superficially, as well as in in-depth breast ultrasound^24^. However, high frequency ultrasound is physically limited in very obese subjects, especially in the abdominal setting. Similarly, in a head-to-head approach visualization of dermis vascularization has previously been shown to be improved with the use of a novel vascular imaging technique compared to power-Doppler^25^. In abdominal ultrasound, new software (e.g. image fusion) implemented in ultrasound aiming at optimized conventional B-scan ultrasound in combination with the use of high-frequency probes has shown to lead to a better diagnostic accurancy for lesions in parenchymatous organs^26^. It is such clinical use that exemplifies the benefit of a thoughtful approach to technologies aiming at higher ultrasound imaging quality.

Limitations of our study include a relatively small sample size and a monocentric approach. Notably, addressing this alongside here presented definition of ultrasound imaging quality, it needs to be considered that the current trial represents a pilot study with limited previous work to refer to. Thus, despite mentioned limitations, our study sets the stage for ongoing research in this field aiming at a definition of abdominal ultrasound quality and diagnostic minimum imaging quality standards for here performed examinations. Ultrasound quality scoring was dependent on the investigator. This effect was attenuated by implying a wide range of items in our score. Our study further primarily focused on differences between a standard probe and high-performance ultrasound probes. Differences in software presets within the two high-performing probes may however have influenced the within-comparison of the latter. Our study cannot provide preset parameters for ultrasound probes to reduce a detrimental effect of obesity on imaging quality, an effort that future studies most certainly will have to address to provide further progress in this field.

In conclusion, imaging quality of liver and kidney, defined as the ability to investigate a multitude of crucial anatomic structures of these organs to visually asses these organs as a whole, deteriorates with greater body size and is independent of the type of ultrasound probe, i.e. standard versus high-performance, used. In subjects with severe obesity, deterioration of liver and kidney assessment is affected by probe performance, that is, high-performance ultrasound probes reduced the detrimental effect of body size on imaging quality. In order to provide a good standard of care to subjects with obesity, here presented results demand for the implementation of imaging quality standards in the investigation of patients with relevant obesity and call for a critical evaluation of the suitable imaging technique in routine and preventive ultrasound diagnostics.

### Supplementary Information

Supplementary Information.

## Data Availability

The study data are available on request. Please contact Thomas Karlas (thomas.karlas@medizin.uni-leipzig.de).
