# Supplementary material for: The application of high-performance ultrasound probes increases anatomic depiction in obese patients
Source: Sci Rep. 2023 Sep 28;13:16297. doi: 10.1038/s41598-023-43509-9 (PMC10539468; doi:10.1038/s41598-023-43509-9)
Supplement: Supplementary file 1 — Supplementary Information. [file 41598_2023_43509_MOESM1_ESM.docx]

# Supplement material

**The application of high-performance ultrasound probes increases anatomic depiction in obese patients.**

Sascha Heinitz^1,2^, Jürgen Müller^3^, Klaus-Vitold Jenderka^4^, Haiko Schlögl^1,2^, Michael Stumvoll^1,2^, Matthias Blüher^1,2^, Valentin Blank^5,6#^, Thomas Karlas^5#*^

**#VB and TK contributed equally to this work.**

* corresponding author

## Ultrasound probes

Compared to the standard probe **(SP)**, high-performance probe **(HPP) 1** will add crystal elements depending on the depth of the area of interest to generate ultrasound waves that would allow for a more focused assessment of tissues up to 400 mm (in an updated software version even up to 550 mm, not used in this study). **HPP2** relies on individual control of matrix elements, thus addressing phase and amplitude issues, as well as signal-to-noise ratio in the far field. Additionally, pulses are paralleled to generate precise acoustic fields and the application of a wideband single crystal array technology allows the transmission and reception of wide band pulses to achieve high signal-to-noise ratio at maximum resolution for high and low frequencies. For technical details, please refer to **Supplemental Tab. 1**. For SP and HPP1, standard settings for ultrasound examination were: preset abdomen adjusted by manufacturer, frequency for fundamental ultrasound 6 MHz, receiver 60 dB, all focus in mid-position. For HPP2, standard settings were: preset abdomen also adjusted by manufacturer, frequency for fundamental ultrasound 5.5 MHz, receiver 70 dB, all focus in mid-position. For the application in colour Doppler mode, the medium frequency setting was used for all probes and the PRF settings were adapted for a maximum flow velocity of 20 cm/s.

## Liver phantoms

Phantom 1 (GAMPT VK-10420, GAMPT mbH, Merseburg, Germany), made of polyurethane with a speed of sound ~1460 m/s, includes vertical, horizontal, and axial-lateral targets aligned in multiple groups. Phantom 1 was used to illustrate differences in imaging quality at plain sight.

Phantom 2 (CIRS ATS 539, Norfolk, VA) consists of polyvinylchloride (speed of sound: 1450 m/s, attenuation coefficient 0.5 dB/cm/MHz at 3.5 MHz). It includes: i) non-echogenic, cylindrical targets (cysts, depth from 65 to 108 mm; maximal number of cysts counted in three rows), ii) axial-lateral targets (dots, 153 to 172 mm; maximal number of targets detected. Detection of cysts, axial discrimination, and distance measurements were analyzed comparing SP versus HPP1 and HPP2.

The attenuation coefficient of phantom1 was not provided, but is in the range of 0.8 to 1.2 dB/cm/MHz as per comparison with phantom 2.

## Ultrasound examination and ultrasound quality scores

### Liver ultrasound score

The liver of study participants was examined using a highly standardized approach via B-mode ultrasound, duplex, and elastography in order to generate a custom ultrasound quality score for comparison among probes. The score considered B-mode ultrasound assessment of arterial, venous, and biliary structures within the liver, organ size and contour, the ability to visually assess the organ as a whole, as well as hepatic parenchyma in pre-specified areas. Duplex mode was used to depict vascularization of the organ. B-mode and duplex assessment was rated as based on quality, which then entered the overall ultrasound quality score. Maximal scoring for optimal imaging quality were 25 points.

### Kidney ultrasound score

Organ size and contour were assessed using B-mode imaging. Arterial vascularization of the organ was investigated via duplex imaging. For better readability and as only the right kidney was investigated in present study, the right kidney will be referred to as kidney from now on. Maximal scoring for optimal assessment were 6 points

### Vascular ultrasound score

Arterial and venous vascularization of the liver and right kidney were investigated via duplex imaging. Maximal scoring for optimal vascular assessment quality were 17 points.

A detailed description of investigated structures and how ultrasound scores were calculated can be found in **Supplemental Tab. 1**.

## Elastography, liver stiffness measurements, and controlled attenuation parameter

Transient elastography allowed for assessment of liver stiffness (in kPA). Elastography measurements were performed immediately before or after the ultrasound study examinations following general recommendations for liver stiffness measurements[1].

As elastography reference, liver stiffness (in kPa) and controlled attenuation parameter (CAP, in dB/m) were measured using a Fibroscan Compact 530 device equipped with M and XL probes as previously described (vibration-controlled transient elastography, VCTE)[2]. A liver stiffness value of ≥8 kPa defined the risk of clinically relevant fibrosis[3]. Steatosis grade was estimated according to the CAP value using previously defined cut-offs for patients at risk for nonalcoholic fatty liver disease (NAFLD[4].

Shear-wave elastography (SWE) was performed using SP, HPP1 and HPP2. The SP and HPP1 are designed for point-SWE, which was performed according to previously published recommendations[5]. In line with Fibroscan, 10 valid measurements (median valid) with an inter-quartile range (IQR) <30% indicated a reliable measurement which was expressed in m/s. A cut-off of 1.32 m/s indicated the risk of relevant fibrosis[6]. High-performance probe 2 is designed for 2D-SWE. Measurements were performed as previously published (unit: kPa; median value of 10 valid single shots, fibrosis cut-off 8 kPa)[7].

**Supplemental figures**

**
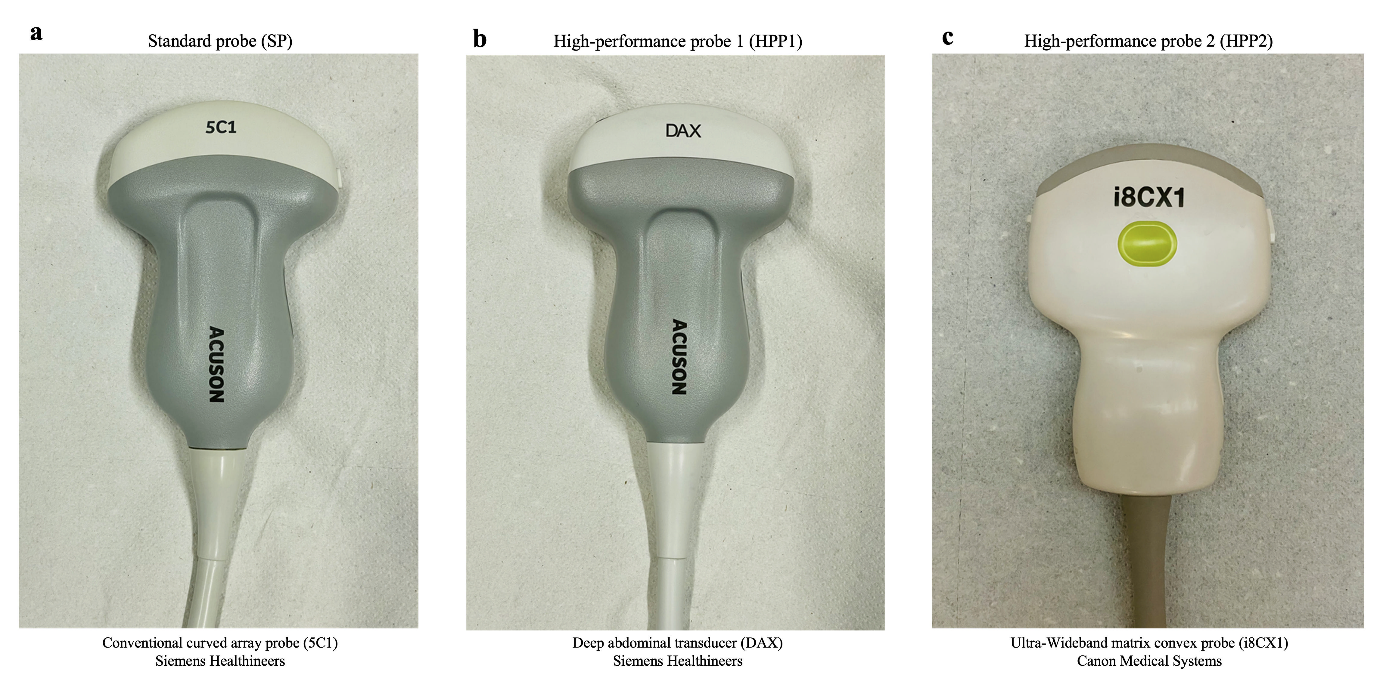
**

**Supplemental figure 1. Illustration of ultrasound probes used for investigation of abdominal imaging quality. (a)** Standard probe (SP) – conventional curved array probe (5C1) by Siemens Healthineers; **(b)** High-performance ultrasound probe 1 (HPP1) – Deep abdominal transducer (DAX) by Siemens Healthineers; **(c)** High-performance ultrasound probe 2 (HPP2) – Ultra-Wideband single crystal matrix convex probe (i8CX1) by Canon Medical Systems.

**
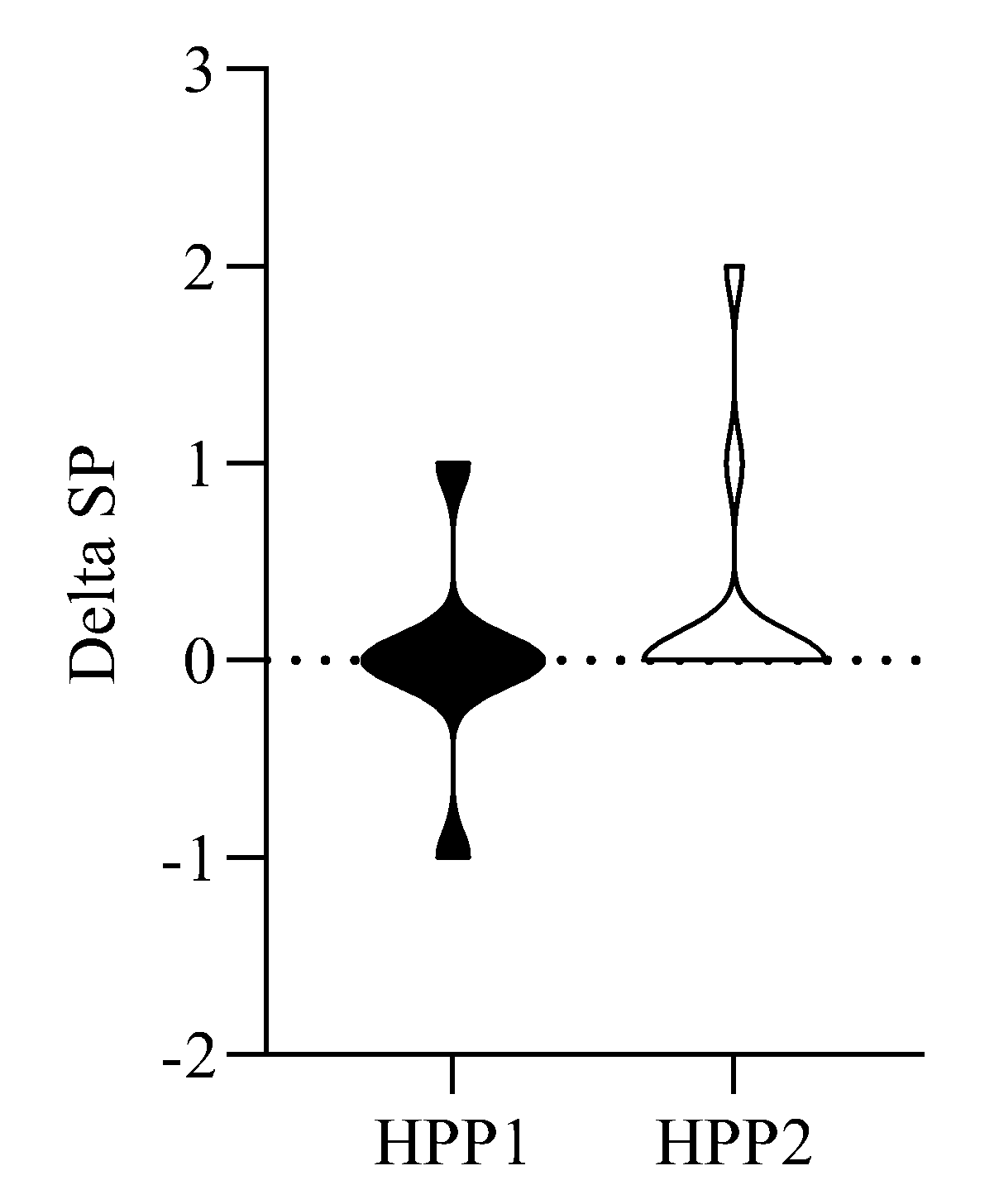
**

**Supplemental figure 2. Scoring for the degree of steatosis hepatis.**

Use of HPP1 led to comparable scores compared to SP, whereas HPP2 tended to overestimate hepatic fat infiltration. Delta SP calculated as SP minus HPP1 and HPP2, respectively. Abbreviations: HPP1, high-performance probe 1; HPP2, high-performance probe 2; SP, standard probe

**
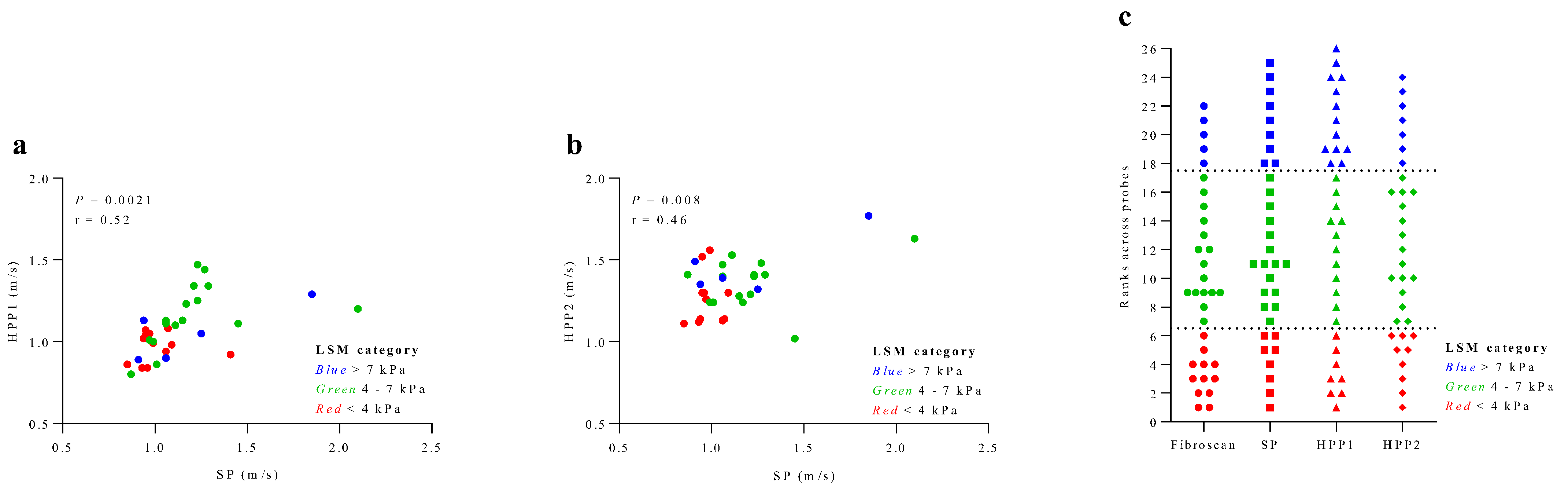
**

**Supplemental figure 3. Correlation of measures of elastography and elastography ranking relative to Fibroscan across probes.**

Liver elastography, as assessed using HPP1 and HPP2, correlated with measurements performed with SP (all *P* < 0.05, A and B). In the normal range of tissue stiffness (defined by LSM > 4 kPa [rank 6] and < 7 kPa [rank 18], green symbols), rank distribution across probes varies considerably (C). Notably, in few subjects liver stiffness > 7 kPa (blue symbols) was measured. Abbreviations: HPP1, high-performance probe 1; HPP2, high-performance probe 2; LSM, liver stiffness measurement; SP, standard probe.

**Supplemental tables**

**Supplemental Table 1.**

Probes used to assess imaging quality. Abbreviations: HPP1, high-performance probe 1; HPP2, high-performance probe 2; SP, standard probe.

| **Probe** | **SP** | **HPP1** | **HPP2** |
| --- | --- | --- | --- |
| Manufacturer | Siemens Healthineers (Erlangen, Germany) | Siemens Healthineers | Canon (Ota, Japan) |
| Technique | Single crystal curved 1D | Multi-D Piezoceramic | Wideband multi-frequency single crystal matrix array transducer |
| Bandwith | Bandwith 1.0–5.7 MHz | Bandwith 1.0–3.5 MHz | Bandwith 1.8–6.4 MHz |
| Field of view | Field of view 72° | Field of view 50° | Field of view 70° |
| Axial/lateral resolution | 0.67/1.2 mm | 0.8/2.3 mm | 0.8/2.3 mm |

**Supplemental Table 2.** Items included in ultrasound quality scores. Maximal scoring as indicated, one point per item (y, yes; n, no). Items included in total versus subscores as highlighted. Abbreviations: T, total ultrasound score; L, liver ultrasound score; K, kidney ultrasound score; V, vascular ultrasound score.

| **Scores** | **T**  33 points | **L**  25 points | **K**  6 points | **V**  17 points |
| --- | --- | --- | --- | --- |
| **B-mode assessment** |  |  |  |  |
| *Hepatic veins* |  |  |  |  |
| Origin of hepatic veins discernable? y/n | x | x |  | x |
| Contour of one hepatic vein discernable for a distance ≥ 1 cm? y/n | x | x |  | x |
| Peripheral imaging of hepatic vein 2 cm below liver capsule for a distance ≥ 5 cm? y/n | x | x |  |  |
| Contour and course of at least one hepatic vein discernable for a distance ≥ 5 cm? y/n | x | x |  |  |
| *Portal vein* |  |  |  |  |
| Division of portal vein within the liver detectable? y/n | x | x |  | x |
| Left and right branch of the portal vein detectable 2 cm beyond division of portal vein within the liver? y/n | x | x |  | x |
| *Liver hilum* |  |  |  |  |
| Portal vein  Good assessment – Contour discernable, anechoic lumen? y/n  Decent assessment – Contour not discernable, hypoechoic lumen? y/n  Poor assessment – Contour/lumen not detectable? y/n | x | x |  | x |
| Detection of ductus hepatocholedochus? y/n | x |  |  |  |
| *Liver parenchyma* |  |  |  |  |
| Liver parenchyma and recessus of liver entirely assessed? y/n | x | x |  |  |
| Measurement of liver size possible? y/n | x | x |  |  |
| Assessment of liver parenchyma at the origin of hepatic veins? y/n | x | x |  |  |
| Assessment of liver parenchyma at the division of the portal vein? y/n | x | x |  |  |
| Assessment of liver parenchyma for a distance ≥ 5 cm along the middle hepatic vein? y/n | x | x |  |  |
| *Extrahepatic structures* |  |  |  |  |
| Subcostal detection of diaphragm at the dorsal recessus? y/n | x | x |  |  |
| Assessment of organ contour?  Fully assessable? y/n  Partly assessable? y/n  Not to be assessed? y/n | x | x |  |  |
| *Assessment of steatosis possible? y/n* | x | x |  |  |
| *Right kidney* |  |  |  |  |
| Assessment of craniocaudal distance possible? y/n | x |  | x |  |
| Full assessment of parenchyma possible? y/n | x |  | x |  |
| Renal artery detectable? y/n | x |  | x |  |
| **Duplex sonography** |  |  |  |  |
| Transcostal assessment of right hepatic vein 2 cm prior to origin  Linear? y/n  Intermittent? y/n  None? y/n | x | x |  | x |
| Assessment of portal vein at liver hilum  Linear? y/n  Intermittent? y/n  None? y/n | x | x |  | x |
| Transcostal assessment of hepatic artery  Linear? y/n  Intermittent? y/n  None? y/n | x | x |  | x |
| Assessment of renal artery possible? y/n | x |  | x | x |
| Calculation of renal resistive index possible? y/n | x |  | x | x |
| **Shear wave elastography** |  |  |  |  |
| Shear wave elastography possible (10 measurements versus abort)? y/n | x | x |  |  |
| *Investigator’s evaluation* |  |  |  |  |
| Highly probable that a lesion ≥ 1 cm would have been detected? y/n | x | x |  |  |
| Highly probable that thrombosis of the portal vein would have been excluded? y/n | x |  |  | x |

**Supplemental table 3.** Values reported as mean ± SD. Abbreviations: BMI, body mass index; CAP, controlled attenuation pattern; HPP1/2, high-performance ultrasound probe 1/2; standard probe; WHR, waist-to-hip

| **Optimal imaging quality for the total ultrasound score (≥ 32 points)** | | | |
| --- | --- | --- | --- |
|  | SP (n = 8) | HPP1 (n = 3) | HPP2 (n = 9) |
| BMI (kg/m^2^) | 26.3 ± 6.7 | 25.2 ± 9.3 | 25.9 ± 6.4 |
| WHR | 0.85 ± 0.18 | 0.80 ± 0.05 | 0.83 ± 0.17 |
| CAP (dB/m) | 212.5 ± 46.0 | 221.7 ± 81.9 | 203.1 ± 51.7 |
| **Non-optimal imaging quality for the total ultrasound score (< 32 points)** | | | |
|  | *SP* (n = 32) | *HPP1* (n = 37) | *HPP2* (n = 31) |
| BMI (kg/m^2^) | 35.6 ± 8.8 | 34.4 ± 8.9 | 36.0 ± 8.6 |
| WHR | 0.91 ± 0.13 | 0.91 ± 0.14 | 0.92 ± 0.13 |
| CAP (dB/m) | 277.8 ± 75.5 | 266.0 ± 73.9 | 284.0 ± 70.1 |

**Supplemental literature**

1. Dietrich CF, Bamber J, Berzigotti A et al. EFSUMB guidelines and recommendations on the clinical use of liver ultrasound elastography, update 2017 (short version). Ultraschall in Med 2017; 38: 377-394.

2. Blank V, Petroff D, Beer S et al. Current NAFLD guidelines for risk stratification in diabetic patients have poor diagnostic discrimination. Scientific reports 2020; 10: 1-11.

3. Baba H, Bahr M, Bernatik T et al. Updated S2k Clinical Practice Guidelines on Non-alcoholic Fatty Liver Disease (NAFLD) issued by the German Society of Gastroenterology, Digestive and Metabolic Diseases (DGVS).

4. Petroff D, Blank V, Newsome PN et al. Assessment of hepatic steatosis by controlled attenuation parameter using the M and XL probes: an individual patient data meta-analysis. The Lancet Gastroenterology & Hepatology 2021; 6: 185-198.

5. Karlas T, Pfrepper C, Wiegand J et al. Acoustic radiation force impulse imaging (ARFI) for non-invasive detection of liver fibrosis: examination standards and evaluation of interlobe differences in healthy subjects and chronic liver disease. Scandinavian journal of gastroenterology 2011; 46: 1458-1467.

6. Cassinotto C, Boursier J, de Lédinghen V et al. Liver stiffness in nonalcoholic fatty liver disease: a comparison of supersonic shear imaging, FibroScan, and ARFI with liver biopsy. Hepatology 2016; 63: 1817-1827.

7. Ronot M, Ferraioli G, Müller H-P et al. Comparison of liver stiffness measurements by a 2D-shear wave technique and transient elastography: results from a European prospective multi-centre study. European radiology 2021; 31: 1578-1587.
